# Hypofractionated and stereotactic body proton radiotherapy for a high-risk lung cancer cohort: A multi-institutional experience from the proton collaborative group prospective registry

**DOI:** 10.1016/j.ctro.2026.101291

**Published:** 2026-09-15

**Authors:** Alexander J. Allen, Charles B. Simone, Kai Sun, David F. Alicia, Ajay Banskota, Gopal K. Bajaj, John H. Chang, James R. Gray, Ryan S. Grover, Christian C. Hyde, Jenna B. Jatczak, Kara E. Lehman, Mark W. McDonald, Stephen A. Mihalcik, Sina Mossahebi, Michelle R. Mundis Myers, Yosan G. Okbamichael, Stephanie M. Perkins, Robert H. Press, Zaker H. Rana, Lane R. Rosen, Ronny L. Rotondo, Christopher C. Sinesi, Henry K. Tsai, James J. Urbanic, Carlos E. Vargas, Nathan Y. Yu, Jing Zeng, Matthew J. Ferris

**Affiliations:** a Department of Radiation Oncology, University of Maryland School of Medicine, Baltimore, MD, United States of America; b Maryland Proton Treatment Center, Baltimore, MD, United States of America; c Memorial Sloan Kettering Cancer Center, Department of Radiation Oncology, New York, NY, United States of America; d Inova Schar Cancer Institute, Department for Advanced Radiation Oncology and Proton Therapy, Fairfax, VA, United States of America; e Oklahoma Proton Center, Oklahoma City, OK, United States of America; f Tennessee Oncology Proton Center, Nashville, TN, United States of America; g Covenant Health, Thompson Proton Center, Knoxville, TN, United States of America; h McLaren Proton Therapy Center, Flint, MI, United States of America; i Emory Proton Therapy Center, Atlanta, GA, United States of America; j Northwestern Feinberg School of Medicine, Department of Radiation Oncology, Chicago, IL, United States of America; k Washington University School of Medicine, Department of Radiation Oncology, St. Louis, MO, United States of America; l Baptist Health South Florida Miami Cancer Institute, Miami, FL, United States of America; m Willis Knighton Health, Shreveport, LA, United States of America; n University of Kansas Medical Center, Department of Radiation Oncology, Kansas City, KS, United States of America; o Hampton University Proton Cancer Institute, Hampton, VA, United States of America; p ProCure Proton Therapy Center, Somerset, NJ, United States of America; q University of California San Diego Health, Department of Radiation Oncology, Encinitas, CA, United States of America; r Mayo Clinic Comprehensive Cancer Center, Department of Radiation Oncology, Phoenix, AZ, United States of America; s University of Washington Medical Center, Department of Radiation Oncology, Seattle, WA, United States of America

**Keywords:** proton beam therapy, SBRT, moderately hypofractionated RT, Lung cancer, Non-Small Cell Lung Cancer, ablative radiotherapy, ultracentral, re-irradiation, Proton therapy, Intensity-Modulated Proton Therapy, pencil-beam scanning, 4DCT

## Abstract

**Introduction:**

Lung cancer is commonly treated with stereotactic body radiotherapy (SBRT) and hypofractionated RT (HFRT). Delivering these ablative regimens with proton beam therapy (PBT) may yield dosimetric and clinical advantages, particularly for high-risk cases. We evaluated safety and efficacy of SBRT/HFRT PBT for lung tumors, including unfavorable subgroups.

**Methods:**

Adults with lung tumors treated with PBT ≤15 fractions were identified from a prospective, multi-institutional registry. Eligible regimens included 4–5 fractions at ≥10Gy/fraction (≥48Gy total) or 8–15 fractions at ≥3.5Gy/fraction (≥52.5Gy total). Local control (LC) was estimated using cumulative incidence with death as a competing risk, and Fine-Gray models were used for subgroup comparisons.

**Results:**

104 patients with 110 tumors (83.6% non-small cell lung cancer) were included: 32.7% ≥4 cm, 41.8% ultracentral, and 30% re-irradiated. Most common regimens were 60Gy/15 fractions (40%) and 50Gy/5 fractions (17.2%); 71.8% received intensity-modulated PBT. 1-and 2-year LC rates were 83.1% and 77.4%, with no difference by tumor size, ultracentral location, or re-irradiation. Internal gross tumor volume receiving 95% of dose was associated with improved LC (*p* = 0.007). Most common toxicities were cough (40%) and dyspnea (34.5%), with no Grade ≥ 3 events.

**Conclusions:**

In the largest prospectively enrolled report of SBRT/HFRT via intensity-modulated PBT for a high-risk lung tumor cohort, 1- and 2-year LC rates were 83.1% and 77.4%, with no grade ≥ 3 toxicities. LC was similar among high-risk subgroups. However, short survival and follow-up durations limited assessment of the findings. Despite the prospective registry, reliable long-term follow-up data remained limited for many patients, somewhat restricting result interpretation.

## Highlights

• Proton SBRT/HFRT showed 83% and 77% 1- and 2-year local control.
• Cohort of high-risk lung tumors with median OS of 12.5 months.
• Ultracentral, ≥4 cm, or re-irradiated tumors had similar local control.
• Cough and dyspnea most common toxicities with no G3+ events.
• Greater IGTV coverage was associated with improved local control.

## 1 Introduction

For patients with localized lung cancer, ablative radiation therapy (RT) dose-fractionation schemes have been established as key options for curative management. Stereotactic body radiation therapy (SBRT), typically defined as RT given over 1–5 treatments at a high dose per fraction, allows for a tumoricidal dose to the target structure with sharp fall-off to minimize adjacent normal tissue dose [1]. High quality data have demonstrated lung SBRT is associated with excellent tumor control rates and favorable safety profiles [1], [2], [3], [4]. However, delivering SBRT to tumors near sensitive mediastinal organs is associated with significant toxicity risks [4]. Hypofractionated radiotherapy (HFRT), delivered in 8–15 fractions, was developed as an alternative to SBRT in these scenarios [5], [6], [7], [8]. However, even with these HFRT techniques, delivering ablative doses with robust target volume coverage can be challenging due to need to respect organ-at-risk (OAR) constraints, especially for central, large, or previously radiated tumors. Ablative (or near-ablative) RT to tumors with these high-risk features can contribute to an increased risk of high-grade toxicities, including treatment-related death [7], [8]. Conversely, sacrificing dose or target volume coverage in the interest of OAR sparing and toxicity risk reduction can contribute to local failure (LF) [9].

Delivering these conformal, high-dose RT regimens via proton beam therapy (PBT) has been explored as an option to safely treat lung tumors bearing adverse clinical and anatomic features [10]. The lack of exit dose and flat dose distribution achievable with PBT potentially allow for greater target volume coverage while more easily meeting OAR constraints for such cases [11]. Multiple investigations have demonstrated excellent oncologic outcomes and toxicity profiles when using proton-based SBRT or HFRT to treat lung cancer [11], [12], [13], [14], [15], [16], [17], [18], [19], [20], [21], [22], [23], [24], [25], [26], [27], [28]. Several of these studies have validated the efficacy of ablative PBT in the treatment of lung cancers with adverse clinical risk factors (RFs) such as large size or central location [15], [16], [17], [18], [19], [20], [21], [22], [23], [24], [25], [26], [27], [28].

However, gaps in the current literature limit its applicability to modern scenarios. Most data evaluating ablative PBT in lung cancer, including all published prospective trials, is based on treatment with older passive scatter (PS) proton technology. Today, 3rd generation PBT using intensity-modulated proton therapy (IMPT) is widely used. Compared to PS, IMPT allows for more modulation of dose distribution, yielding potential for improved target coverage with reduced dose to adjacent tissues [29], [30]. In addition to being predominantly from the PS era, the existing literature does not account for more modern sub-categorizations of central tumors, as defined by recent photon-based HFRT trials such as SUNSET and Nordic-HILUS [7], [8]. Lung tumors in previously re-irradiated fields represent another contemporary high-risk category for which there is sparse evidence validating proton SBRT and HFRT. With this study, we sought to add to the current data assessing proton SBRT and HFRT in lung cancer by describing our analysis of a modern, high-risk cohort from a prospective, multi-institutional registry. We aim to provide an up-to-date assessment of the clinical efficacy and safety of ablative PBT while stratifying for clinically significant categories of high-risk disease.

## 2 Methods

### 2.1 Patient cohort

We assessed consecutive adult patients ≥18 years old who were prospectively enrolled in the Proton Collaborative Group (PCG) multi-institutional registry of proton therapy centers in the United States who received ≤15 fractions of PBT between 1/1/2012 and 6/6/2024 for lung tumors. For patients treated with regimens of 4–5 fractions, only those treated at ≥10 Gy per fraction and to a total dose ≥48 Gy were included. For patients treated with regimens of 8–15 fractions, only those treated at ≥3.5 Gy per fraction and to a total dose of ≥52.5 Gy were included. Patients who received PBT for primary lung tumors of any histology and disease stage were included.

### 2.2 Data collection

Patient demographic and clinical data, oncologic outcomes data, and toxicity data for patients were collected by each participating institution and obtained from the PCG registry for analysis. All toxicities were reported in accordance with the Common Terminology Criteria for Adverse Events (CTCAE). Acute toxicities were defined as those occurring during RT or within ≤90 days of RT completion, while late toxicities were defined as those occurring >90 days after RT completion. RT plans were delivered in accordance with each participating department's institutional protocols. Patient RT treatment plans were obtained via Digital Imaging and Communications in Medicine (DICOM) files (requested from the participating site by PCG and then securely transferred to our institution) and reviewed individually to measure tumor size, determine tumor centrality, and collect OAR and target dose data. Central tumors were defined as gross tumor volume (GTV) located within a 2 cm radius of the following structures: esophagus, great vessels (aorta, pulmonary veins, pulmonary arteries), vertebral bodies, carina, trachea, proximal bronchial tree, heart. Tumors were considered ultracentral if the GTV abutted any of these structures. Tumors were also categorized by whether they met the stricter definition of ultracentral per the SUNSET trial: GTV abutting the proximal bronchial tree, esophagus, pulmonary veins, or pulmonary arteries [6].

### 2.3 Study design

This study is IRB-approved through the REG001–09 prospective registry sponsored by the Proton Collaborative Group (ClinicalTrials.gov ID NCT01255748) to collect and analyze information from patients treated with various forms of RT. This study was conducted in accordance with the Declaration of Helsinki (as revised in 2013). Patients from this registry who received PBT via SBRT and HFRT techniques for lung tumors were identified, and their local control (LC) outcomes and toxicity profiles were analyzed. Patients who received PS or IMPT were included. Cumulative incidence plots of LF with death as a competing risk were created for the entire cohort and for tumor subgroups. Fine-Gray analysis was used to compare cumulative LF incidence between subgroups. Univariate cox regression analysis (UVA) was performed to identify associations of different clinical and treatment characteristics with clinical outcomes. Multivariate cox regression analysis (MVA) was performed with any variables that showed statistically significant associations with LC on UVA. Toxicity incidences were compared using two-sample proportions tests and Chi-Square tests. SPSS software was used for statistical analysis (IBM Corporation, Armonk, NY, USA).

## 3 Results

### 3.1 Sample characteristics

Patient and tumor characteristics are represented in Table 1. The cohort included 110 tumors from 104 patients (median age 73) who were treated across 14 different centers. Most patients were White, with a balanced sex distribution. With regards to performance status, 28.2%, 52.7%, and 15.5% of the cohort had Eastern Cooperative Oncology Group performance status (ECOG-PS) scores of 0, 1, and 2, respectively. Most patients previously smoked but quit (70.2%); however, 18.2% of patients were actively smoking during treatment. The most common tumor histologies were primary lung squamous cell carcinoma (32.7%), primary lung adenocarcinoma (30.0%) and non-small cell lung cancer (NSCLC) not-otherwise specified (19.1%). Other histologies, such as small-cell lung cancer, large-cell lung cancer, and lung sarcomatoid carcinoma, were also represented. Notably, 32.7% of the treated tumors were ≥ 4 cm in their largest dimension, and 37.2% of the tumors were manifestations of recurrent, previously treated disease. Most tumors were centrally located (76.4%), defined as GTV located within 2 cm of a central structure. Peripheral tumors accounted for 8.2% of the sample and most tumors (63.6%) were ultracentral. Additionally, 41.8% of the tumors met the narrower definition of ultracentral location used in the SUNSET trial. DICOM imaging was requested for all eligible patients through PCG and was successfully obtained and analyzed for 85% of patients. For the remaining 15%, imaging could not be obtained despite multiple requests to participating institutions, most commonly because sites were no longer active or did not respond to requests for image transfer. Consequently, tumor location was unavailable for these patients.

**Table 1:** Demographic and Clinical Characteristics.

Number of Tumors	110
Number of Patients	104
Male – no. (%)	57 (51.8)
Female – no. (%)	48.2 (48.2)
Median age – years [IQR]	73 [67–80]
Median Follow-up time – months [IQR]	14.5 [6.25–21.75]
Race – no. (%)	
White	86 (78.2)
Black	11 (10.0)
Asian/Pacific Islander	6 (5.5)
Other	1 (0.9)
Not specified	6 (5.5)
ECOG performance status score – no. (%)	
0	31 (28.2)
1	58 (52.7)
2	17 (15.5)
3	1 (0.9)
4	0 (0.0)
Not specified	3 (2.7)
Smoking History – no. (%)	
Never	12 (10.9)
Yes, quit	78 (70.9)
Yes, current	20 (18.2)
Tumor Histology – no. (%)	
Lung squamous cell carcinoma	36 (32.7)
Lung adenocarcinoma	33 (30.0)
Non-small cell lung cancer, NOS	21 (19.1)
Not biopsied	5 (4.5)
Small-cell lung cancer	4 (3.6)
Large-cell lung cancer	2 (1.8)
Lung sarcomatoid carcinoma	2 (1.8)
Neuroendocrine carcinoma, grade 4	2 (1.8)
Poorly differentiated carcinoma, NOS	2 (1.8)
Carcinoid tumor	1 (0.9)
Malignant spindle cell neoplasm	1 (0.9)
Primary lung melanoma	1 (0.9)
Tumor Location – no. (%)	
Peripheral	9 (8.2)
Central^1^	84 (76.4)
Not specified	17 (15.5)
Ultracentral^2^	70 (63.6)
Ultracentral per SUNSET criteria^3^	46 (41.8)
Median tumor size in greatest dimension – cm [IQR]	2.5 [2.1–4.4]
Tumors greater than or equal to 4 cm in greatest dimension – no. (%)	36 (32.7)
Received prior systemic therapy for this cancer – no. (%)	34 (30.9)
Received prior thoracic surgery for this cancer – no. (%)	19 (17.3)
Current radiotherapy treatment for recurrent cancer – no. (%)	41 (37.2)

Abbreviations: IQR interquartile ratio

ECOG – Eastern Cooperative Oncology Group

NOS - Not otherwise specified

1. Defined as macroscopic disease within 2 cm of central structures (Great vessels, proximal bronchial tree, trachea, esophagus, heart, vertebral bodies)

2. Defined as macroscopic disease abutting any central structures

3. Defined as macroscopic disease abutting the proximal bronchial tree, esophagus, pulmonary arteries, or pulmonary veins.

### 3.2 Radiation therapy characteristics

Of the treated tumors, 30.0% had prior in-field RT (median dose 55 Gy, median 10 fractions) and 3.6% had 2 courses of prior in-field RT **(**Table 2**).** IMPT and PS were used in 71.8% and 27.2% of the treatments, respectively. The median prescribed dose and biologically effective dose (BED) were 60 Gy and 89.8 Gy, respectively. The most frequent dose-fractionation regimens were 60 Gy in 15 fractions (40%), 50 Gy in 5 fractions (17.2%), 70 Gy in 10 fractions (10%), and 60 Gy in 8 fractions (7.2%). The median relative maximum dose (Percent D_max_) was 107% of the prescribed dose and median absolute D_max_ was 63.9 Gy. The median percent volume receiving 95% of the prescription dose (V95) of the internal GTV (IGTV) was 100%, and the median dose received by 90% of the IGTV (D90) was 60.1 Gy. Median dose to 0.03 cc of the volume (D0.03) to the heart, great vessels, proximal bronchial tree, and esophagus were 24.1, 48.8, 36.6, and 5.3 Gy, respectively. Regarding motion management techniques, approximately 41% of tumor treatments were planned using free-breathing four-dimensional computed tomography (4DCT) to track the tumor path during the breathing cycle. In close to 25% of tumors no motion management strategies were implemented. Roughly 5% of tumors were planned using either breath hold, abdominal compression belt, or 4DCT combined with a compression belt. Data on image-guidance paradigms during radiation treatment delivery were not available for analysis.

**Table 2:** Radiotherapy Treatment Characteristics.

Prior in-field radiotherapy – no. (%)	33 (30)
One prior course of in-field radiotherapy – no. (%)	29 (26.4)
Two prior courses of in-field radiotherapy – no. (%)	4 (3.6)
Proton radiotherapy delivery	
Intensity-Modulated Proton Therapy	79 (71.8)
Passive scattering	30 (27.2)
Not specified	1 (0.9)
Median Radiation Dose – Gy [IQR]	60 [54.6–60.1]
Median Number of Fractions of Radiation [IQR]	10 [5–15]
Median Dose per Fraction – Gy [IQR]	4.4 [4–7.5]
Biologically Effective Dose – Gy [IQR]	89.8 [84–100]
Motion Management Techniques	
Free-Breathing 4DCT	45 (40.9)
None	27 (24.6)
Breath Hold	2 (1.8)
Abdominal Compression Belt	1 (0.9)
4DCT + Compression Belt	2 (1.8)
Not Reported	33 (30)
Dose and Fractionation Regimens – no. (%)	
60 Gy in 15 fractions	44 (40.0)
50 Gy in 5 fractions	19 (17.2)
70 Gy in 10 fractions	11 (10)
60 Gy in 8 fractions	8 (7.2)
60 Gy in 10 fractions	5 (4.5)
54 Gy in 15 fractions	5 (4.5)
53 Gy in 15 fractions	4 (3.6)
67 Gy in 15 fractions	3 (2.7)
50 Gy in 4 fractions	2 (1.8)
70 Gy in 14 fractions	1 (0.9)
56 Gy in 14 fractions	1 (0.9)
63 Gy in 15 fractions	1 (0.9)
63 Gy in 10 fractions	1 (0.9)
Median Absolute Maximum Dose – Gy [IQR]	63.9 [61.4–66.2]
Median Relative Maximum Dose – Percent [IQR]	107 [105–110]
Median IGTV V95 – % [IQR]	100 [99.9–100]
Median IGTV D90 – Gy [IQR]	60.1 [53.5–60.9]
Median CTV V95 – % [IQR]	99.8 [96.6–100]
Median CTV D90 – Gy [IQR]	59.7 [53.6–60.8]
Median Doses to Organs at Risk – Gy [IQR]	
Heart D0.03cc	24.1 [13.5–55.3]
Carina D0.03cc	3.8 [3–41.2]
Spinal Cord D0.03cc	2.7 [0.5–14.6]
Great Vessels D0.03cc	48.8 [11.2–60.7]
Proximal Bronchial Tree D0.03cc	36.6 [1.8–60.5]
Esophagus D0.03cc	5.3 [0.2–31.3]
Lung-GTV Mean Dose	3.2 [2.1–6.3]

Abbreviations, IQR: Interquartile ratio

4DCT: Four-Dimensional Computed Tomography.

IGTV: Internal Gross tumor volume.

CTV: Clinical treatment volume.

V95: Percentage of target structure volume receiving 95% of prescribed dose.

D90: Minimum radiation dose received by 90% of the target volume.

D0.03cc Maximum dose received by the smallest 0.03 cm^3^ (cc) of an organ at risk.

### 3.3 Clinical outcomes

**Fig. 1:**
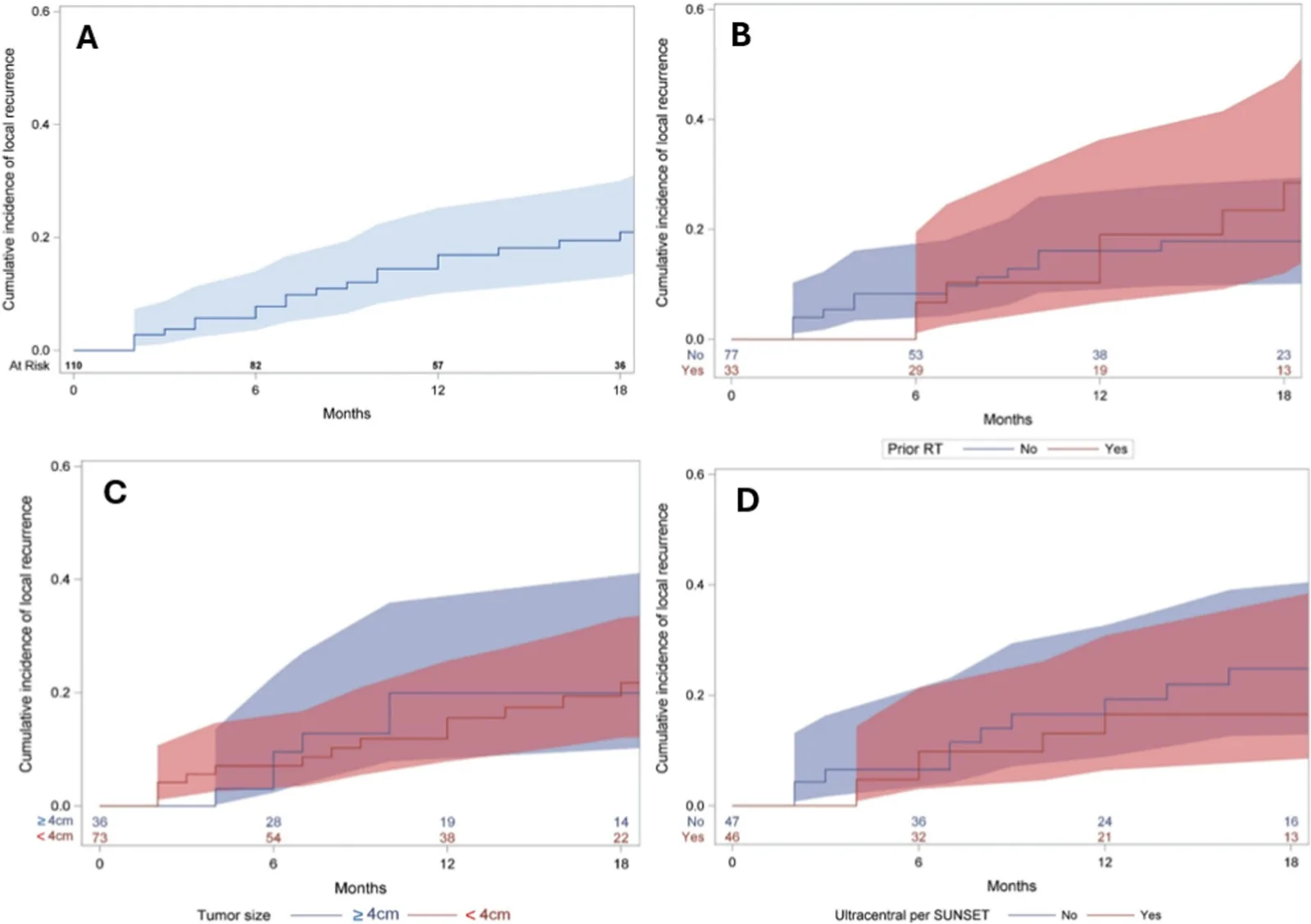
Cumulative incidence plots of local disease recurrence (A) for the entire cohort and stratified by (B) receipt of previous in-field radiation, (C) largest tumor dimension greater than or equal to 4 cm versus less than 4 cm (D) ultracentral location per SUNSET. Cumulative incidence rates were compared via Fine-Gray analysis.

For the entire cohort, MFU time was 14.5 months [IQR 6.25–21.75] and median overall survival (OS) was 12.5 months [IQR 5.75–24]. Based on cumulative incidence functions accounting for death treated as a competing risk, the 1 and 2-year LC rates for the entire cohort were 83.3% and 77.4%, respectively. For tumors in previously irradiated regions, 1 and 2-year LC rates were 83.3% and 82.2% For tumors with largest dimension of ≥4 cm, 1 and 2-year LC rates were 80.1% and 75.8%. For SUNSET-defined ultracentral tumors, 1 and 2-year LC rates were 80.7% and 75.2%. Given the relatively short follow-up duration and limited survival in this cohort, LC estimates beyond 2 years were considered unreliable and are therefore not reported. Fine-Gray analysis revealed no differences in cumulative LF incidence (with death as a competing risk) between any subgroups (Fig. 1A-D**).** UVA showed no statistically significant associations of LC with largest tumor dimension ≥4 cm, recurrent versus de novo disease, receipt of prior in-field RT, total RT treatment time, BED, ultracentral location, proton beam modality, or IGTV D90 **(**Table 3**).** A higher volume of the IGTV receiving 95% of the prescribed dose (IGTV V95) was associated with improved LC (HR 0.982, [95% CI 0.97–0.995], *p* = 0.007) **(**Table 3**)**. Continuing to smoke at the time of treatment was associated with worsened LC to a degree that approached statistical significance (*p* = 0.066).

**Table 3:** Univariate Analysis.

Characteristic	Hazard Ratio	95% Confidence Interval	*P-*value
Tumor greatest dimension ≥4 cm^1^	1.140	[0.49–2.655]	0.761
Recurrent disease^2^	1.644	[0.727–3.715]	0.232
Prior RT in same region	1.656	[0.731–3.752]	0.227
Passive Scatter PBT^3^	1.001	[0.955–1.049]	0.965
Smoking history but quit^4^	2.312	[0.326–16.397]	0.402
Current smoker^4^	6.502	[0.881–47.993]	0.066
Ultracentral location^5^	1.058	[0.396–2.83]	0.910
Ultracentral location per SUNSET^5^	0.640	[0.255–1.607]	0.342
Squamous cell histology^6^	1.70	[0.617–4.899]	0.296
Biologically Effective Dose	1.017	[0.984–1.050]	0.318
Total RT treatment duration	1.001	[0.955–1.049]	0.965
IGTV D90	1.001	[1.0–1.001]	0.088
IGTV V95	0.982	[0.97–0.995]	0.007

1. Tumors with greatest dimension < 4 cm served as the reference

2. Patients with de novo disease served as the reference

3. Tumors treated with received intensity-modulated proton therapy (IMPT) served as the reference

4. Patients with no smoking history served as the reference

5. Defined as any patient tumor abutting central structures (pulmonary arteries, pulmonary veins, heart, esophagus, proximal bronchial tree, vertebrae, mediastinum). Patients with non-ultracentral location served as the reference

6. Tumors with adenocarcinoma histology served as the reference

Overall, the most frequently observed toxicities were cough (40%), dyspnea (34.5%), fatigue (31.8%), radiation dermatitis (RD) (17.3%), esophagitis (5.4%), and dysphagia (5.4%) **(**Table 4**)**. The majority of toxicities were acute (occurring during radiation or within 90 days of RT completion). The most common overall grade 1 (G1) toxicities for the entire cohort were cough, fatigue, and dyspnea at 30%, 27.3%, and 15.5%, respectively. The most common grade 2 (G2) toxicities were dyspnea, cough, and fatigue at 12.7%, 7.3%, and 3.6%, respectively. No grade 3 (G3) or greater toxicities were observed. Toxicity comparisons between subgroups revealed an increased cough among patients treated to areas with prior in-field RT compared to those receiving first-time RT (57.6% versus 32.5%, *p* = 0.014)**.** Increased rate of G2 cough was noted among patients with SUNSET-defined ultracentral tumors versus non-ultracentral (13% versus 2.1%, *p* = 0.046). Additionally, overall incidence of chest wall pain was 8.3% among those with tumor greatest dimension of ≥4 cm, compared to 0.0% for tumors <4 cm (*p* = 0.012). Patients with tumors ≥4 cm also experienced greater overall rates of radiation pneumonitis (RP) compared to those <4 cm (5.6% versus 0%, *p* = 0.042). Otherwise, there were no differences in toxicity incidence by grade among the subgroup comparisons of tumor size ≥4 cm versus <4 cm, ultracentral tumor location versus non-ultracentral, and re-irradiation versus first-time radiation **(**Table 5**)**. When toxicity incidence was compared between PS and IMPT, there was a higher overall incidence of RD (33.3% versus 11.4%, *p* = 0.007) and G1 RD with PS (30% versus 10.1%, *p* = 0.011). PS was also associated with an overall higher incidence of hoarseness (13.3% versus 1.3%, *p* = 0.007) and higher incidence of G1 hoarseness (13.3% versus 0%, *p* = 0.001). Patients treated with PS also demonstrated a higher incidence of G2 fatigue (10% versus 1.3%, *p* = 0.030) **(Supplemental Table 1).**

**Table 4:** Acute and Late Toxicity Incidence.

Acute Toxicity (Onset during PBT or ≤ 90 days after PBT completion), *n* = 110 evaluable			
Toxicity	Grade 1	Grade 2	Grade 3
Cough	31 (28.2%)	5 (4.6%)	0 (0%)
Fatigue	26 (23.6%)	4 (3.6%)	0 (0%)
Radiation Dermatitis	17 (15.5%)	2 (1.8%)	0 (0%)
Dyspnea	16 (14.6%)	12 (10.9%)	0 (0%)
Dysphagia	4 (3.6%)	0 (0%)	0 (0%)
Esophagitis	3 (2.7%)	1 (0.9%)	0 (0%)
Hoarseness	4 (3.6%)	1 (0.9%)	0 (0%)
Chest Wall Pain	2 (1.8%)	0 (0%)	0 (0%)
Radiation Pneumonitis	0 (0%)	0 (0%)	0 (0%)
Laryngeal Hemorrhage	1 (0.9%)	0 (0%)	0 (0%)

Late Toxicity (Onset >90 days after PBT completion), *n* = 102 evaluable			
Toxicity	Grade 1	Grade 2	Grade 3
Cough	2 (2.0%)	3 (2.9%)	0 (0%)
Fatigue	4 (3.9%)	0 (0%)	0 (0%)
Radiation Dermatitis	0 (0%)	0 (0%)	0 (0%)
Dyspnea	1 (1.0%)	2 (2.0%)	0 (0%)
Dysphagia	1 (1.0%)	1 (1.0%)	0 (0%)
Esophagitis	1 (1.0%)	1 (1.0%)	0 (0%)
Hoarseness	0 (0%)	0 (0%)	0 (0%)
Chest Wall Pain	1 (1.0%)	0 (0%)	0 (0%)
Radiation Pneumonitis	1 (1.0%)	1 (1.0%)	0 (0%)
Laryngeal Hemorrhage	0 (0%)	0 (0%)	0 (0%)

**Table 5:** Toxicity Incidence by Subgroup.

Toxicity	Prior RT	No prior RT	p-value	≥4 cm	< 4 cm	p-value	Ultracentral	Not-ultracentral	p-value
Cough	19 (57.6%)	25 (32.5%)	**0.014**	14 (38.9%)	29 (39.7%)	0.933	19 (41.3%)	17 (36.2%)	0.611
G1	13 (39.4%)	20 (26.0%)	0.159	10 (27.8%)	23 (31.5%)	0.69	12 (26.1%)	15 (31.9%)	0.536
G2	4 (12.1%)	4 (5.2%)	0.2	4 (11.1%)	4 (5.5%)	0.289	6 (13.0%)	1 (2.1%)	**0.046**
Dyspnea	14 (42.4%)	24 (31.2%)	0.255	11 (30.6%)	26 (35.6%)	0.6	16 (34.8%)	15 (31.9%)	0.769
G1	6 (18.2%)	11 (14.3%)	0.604	6 (16.7%)	11 (15.1%)	0.829	7 (15.2%)	7 (14.9%)	0.965
G2	6 (18.2%)	8 (10.4%)	0.261	4 (11.1%)	10 (13.7%)	0.704	6 (13.0%)	6 (12.8%)	0.968
Radiation Dermatitis	16 (20.8%)	3 (9.1%)	0.137	5 (13.9%)	14 (19.2%)	0.494	8 (17.4%)	9 (19.2%)	0.826
G1	2 (6.1%)	15 (19.5%)	0.074	4 (11.1%)	13 (17.8%)	0.365	7 (15.2%)	8 (17.0%)	0.813
G2	1 (3.0%)	1 (1.3%)	0.533	1 (2.8%)	1 (1.4%)	0.606	1 (2.2%)	1 (2.1%)	0.988
Dysphagia	2 (6.1%)	4 (5.2%)	0.855	2 (5.6%)	4 (5.5%)	0.987	4 (8.7%)	2 (4.3%)	0.384
G1	2 (6.1%)	3 (3.9%)	0.617	2 (5.6%)	3 (4.1%)	0.734	3 (6.5%)	2 (4.3%)	0.628
G2	0 (0.0%)	1 (1.3%)	0.511	0 (0%)	1 (1.4%)	0.481	1 (2.2%)	0 (0%)	0.309
Esophagitis	2 (6.1%)	4 (5.2%)	0.855	2 (5.6%)	4 (5.5%)	0.987	3 (6.52%)	2 (4.3%)	0.628
G1	1 (3.0%)	3 (3.9%)	0.824	1 (2.8%)	3 (4.1%)	0.728	1 (2.2%)	2 (4.3%)	0.57
G2	1 (1.3%)	1 (3.0%)	0.533	1 (2.8%)	1 (1.4%)	0.606	2 (4.4%)	0 (0%)	0.148
Fatigue	8 (24.2%)	27 (35.1%)	0.264	11 (30.6%)	24 (32.9%)	0.807	13 (28.3%)	18 (38.3%)	0.305
G1	6 (18.2%)	24 (31.2%)	0.161	9 (25.0%)	21 (28.8%)	0.679	9 (19.6%)	17 (36.2%)	0.074
G2	2 (6.1%)	2 (2.6%)	0.374	1 (2.8%)	3 (4.1%)	0.728	3 (6.5%)	1 (2.1%)	0.296
Laryngeal Hemorrhage	1 (3%)	0 (0%)	0.125	0 (0%)	1 (1.37%)	0.481	1 (2.2%)	0 (0%)	0.309
G1	1 (3%)	0 (0%)	0.125	0 (0%)	1 (1.2%)	0.481	1 (1.2%)	0 (0%)	0.309
G2	0 (0%)	0 (0%)		0 (0%)	0 (0%)		0 (0%)	0 (0%)	
Hoarseness	0 (0%)	5 (6.5%)	0.134	2 (5.6%)	3 (4.11%)	0.734	2 (4.4%)	2 (4.3%)	0.982
G1	4 (5.2%)	0 (0%)	0.182	2 (5.6%)	2 (2.7%)	0.462	1 (2.2%)	2 (4.3%)	0.57
G2	0 (0%)	1 (1.3%)	0.511	0 (0%)	1 (1.4%)	0.481	1 (2.2%)	0 (0%)	0.309
Chest Wall Pain	0 (0%)	3 (3.9%)	0.25	3 (8.3%)	0 (0%)	**0.012**	1 (2.2%)	1 (2.1%)	0.988
G1	0 (0%)	3 (100%)	0.25	3 (8.3%)	0 (0%)	**0.012**	1 (2.2%)	1 (2.1%)	0.988
G2	0 (0%)	0 (0%)		0 (0%)	0 (0%)		0 (0%)	0 (0%)	
Radiation Pneumonitis	0 (0%)	2 (2.6%)	0.35	2 (5.6%)	0 (0%)	**0.042**	1 (2.2%)	0 (0%)	0.309
G1	0 (0%)	1 (50%)	0.511	1 (2.8%)	0 (0%)	0.153	0 (0%)	0 (0%)	0.309
G2	0 (0%)	1 (50%)	0.511	1 (2.8%)	0 (0%)	0.153	1 (2.2%)	0 (0%)	

Median OS was 12.5 months [IQR 5.75–24] with 43 total deaths in the cohort. Cause of death was not reported for 53.2% of mortality events. For the remainder of deaths, 19.2% were attributed to progressive cancer, 17% were possibly attributed to progressive cancer, and the remaining 8.5% were attributed to non-malignant causes such as acute cerebrovascular accident and progression of idiopathic pulmonary fibrosis **(**Table 6**)**.

**Table 6:** Causes of Death.

Cause of Death	n (%)⁎
Not reported	25 (53.2)
Progressive malignancy	9 (19.2)
Possibly from progressive malignancy	8 (17.0)
Progressive idiopathic pulmonary fibrosis	3 (6.4%)
Acute cerebrovascular accident	1 (2.1%)
Unspecified non-malignant cause	1 (2.1%)

⁎ (%) signifies percentage of 47 total deaths.

## 4 Discussion

In this multi-institutional registry cohort of patients with high-risk tumors treated with proton HFRT or SBRT, LC was 83% at 1 year and 77% at 2 years, with a median OS of 12.5 months. Although these rates are modestly lower than certain photon series, interpretation of these outcomes requires consideration of the substantial risk profile of the cohort: 63.6% abutted mediastinal structures, 41.8% were ultracentral per SUNSET, 32.7% were ≥ 4 cm, and 30.0% were previously irradiated (26.4% had 1 prior RT course and 3.6% had 2 prior courses). Many tumors in the cohort bore multiple clinical RFs which existing published data have shown to be associated with greater post-RT LF. For example, when considering the LF-associated RFs of size ≥4 cm, central location, prior in-field RT, and smoking during RT, 37.6% of tumors had two of these RFs, 15.1% had three RFs, and 4.3% had four RFs [31], [32], [33], [34]. There were no LF differences after stratifying by size ≥4 cm, receipt of prior infield RT, or ultracentral tumor location, suggesting that proton SBRT/HFRT has a comparative degree of effectiveness when treating these high-risk subgroups. While our observed overall LC values were nominally slightly lower than trials such as HILUS and SUNSET (which both excluded re-irradiation patients), this was likely a product of cumulative RFs compounding LF likelihood in a high-risk cohort, rather than a reflection of radiation modality.

On UVA, only IGTV V95 showed a direct association with LC. This finding possibly suggests IGTV V95 is a protective parameter, and that maximizing the proportion of IGTV receiving prescription dose can reduce LF risk. More specifically, this finding highlights the importance of IGTV coverage when using a more homogenous planning approach such as proton SBRT/HFRT, in contrast to ablative photon treatment plans which typically have a more heterogenous dose distribution. Past central tumor photon SBRT/HFRT trials have typically undercovered target volumes to meet OAR constraints, yet doing so may come with an LC disadvantage that could be mitigated by PBT physical properties. While metrics like conformity index are not always superior for lung cases with PBT versus photon techniques, the lack of exit dose with PBT often allows for superior target coverage while respecting central OAR maximum and 5–10 cc constraints.

As high-grade toxicities are driven by maximum doses to central OARs for central/ultracentral cases, PBT may be an option to achieve better coverage with lower chance of toxicity. PBT plan hotspots are often lower (107% in our series versus 115–120% in comparative photon series). The greater homogeneity of PBT dose distribution compared to photon plans makes PBT apt for treating high-risk and central tumors, where limiting hotspots is of critical importance. However, the optimal degree of dose heterogeneity for ablative lung radiotherapy in general remains an area of ongoing debate. Photon-based SBRT plans are typically designed to achieve a central hot spot within the GTV that is significantly greater than the prescription dose. This is an approach that has been validated by recent clinical data, including the iSABR phase 2 trial which demonstrated that higher central D_max_ was associated with improved LC [35], [36], [37]. While proton plans typically produce homogenous intratumoral dose distributions with robust optimization, they can also be designed with non-uniform dose prescriptions to achieve comparable heterogeneity to photon SBRT plans [38], [39]. Furthermore, clinical outcomes data have suggested comparable LC between proton and photon SBRT, suggesting that intratumoral heterogeneity differences have not translated into a measurable LC disadvantage for PBT [19]. Standardized reporting of intratumoral dose metrics across radiation modalities is ultimately needed to resolve this question definitively.

The observed overall toxicity profile was low, although toxicity reporting could have been affected by short follow-up duration. While there were some toxicity differences by subgroups, our cohort's toxicity burden maintained a similar pattern after stratifying for different high-risk categories. Overall post-RT incidence of cough was significantly greater among re-RT patients, patients with ultracentral tumors had a higher rate of overall G2 cough, and patients with tumors ≥4 cm experienced higher rates of overall chest wall pain and RP. However, the absolute rates of chest wall pain and RP in this subgroup were low (8.3 and 5.6%, respectively). Most of the reported toxicities were acute (during PBT or within 90 days of PBT completion). Given the suboptimal follow-up of our cohort, one can thus conclude from this data that ablative PBT appears to be safe from an acute toxicity standpoint, but long-term safety remains to be characterized.

It is important to emphasize the significant limitations of our study that make these results less generalizable. The relatively short MFU time and OS rates (14.5 and 12.5 months, respectively) seen in our data were probably multifactorial. Partially, the short OS time could reflect the overall poor performance and prognostic characteristics of the cohort, which could have portended a higher mortality risk. Considerable portions of the cohort were being treated for recurrent cancer and had not only received prior systemic therapy but even prior radiation to the treated tumor. The cohort's 29.1% distant or regional nodal recurrence rate, coupled with a median time to non-local progression of just 5.5 months, further corroborates the inherently unfavorable biology represented in this population. While official causes of death were not available for most patients, 36.2% of deaths were likely or possibly attributed to cancer progression. Additional causes such as cerebrovascular accident and progressive idiopathic pulmonary fibrosis were responsible for 11% of deaths. These clinical characteristics, along with the fact that only 104 patients across multiple institutions received ablative PBT (and were thus eligible for study inclusion) over a 12-year period, suggest a highly selected cohort enriched for patients with substantial comorbidity and unfavorable disease biology. This select patient population is thus not likely to be representative of typical early-stage lung cancer cases, further limiting the external validity of our findings. Our reported LC and adverse event rates must also be considered with this context in mind, as development of regional or distant spread alters a patient's disease course and treatment plan. This in turn could have created an underreporting bias regarding documentation of radiation toxicity and tumor recurrence events. Regarding the short MFU time, many proton centers receive referrals for challenging cases, and then patients often return to their local providers soon after treatment. As such, long-term follow-up data are likely under-captured by the PCG datasets, which could have impacted our observed LC and toxicity rates. We did request (and often received) additional follow-up information from the institutions for patients with insufficient data. However, many clinically significant toxicities such as bronchial stenosis and hemorrhage (especially for centrally located tumors) may not manifest until 12–24 months post-treatment. Therefore, we cannot definitively conclude the relative safety of ablative PBT regimens compared to alternative modalities for lung tumors based on this dataset, as the late toxicity rates remain incompletely characterized. Presumably, PBT is considered a valuable tool for these challenging cases by both treating and referring physicians, and it does seem our results support this despite the limitations of this series.

The marked heterogeneity of our cohort is also an important consideration when contextualizing our findings. There was significant variability in the sample with regards to tumor centrality, prior treatments, and disease stage. Additionally, while roughly 80% of tumors were NSCLC, the remainder comprised diverse histologies such as small-cell lung cancer (3.6%), large cell lung cancer (1.8%), high-grade neuroendocrine carcinoma (1.8%), malignant spindle cell neoplasm (0.9%), and primary melanoma of the lung (0.9%). Treatment techniques also varied, with 27% of PBT delivered via older PS technology and 73% with IMPT, reflecting the transitional era from PS to pencil-beam scanning during the study period [40]. Although all treatments met contemporary standards at the time, this variability limits direct comparison to modern, exclusively IMPT-based proton therapy. However, toxicity rates were similar between PS and IMPT **(Supplementary Table 1)** suggesting that inclusion of PS-treated patients did not substantially influence toxicity outcomes. This clinical diversity of our cohort reflects contemporary real-world practice; however, it also complicates interpretation of both efficacy and adverse effect outcomes. Differences in tumor biology, prior treatment exposure, and baseline risk profiles may have influenced outcomes independently of the PBT approach. We attempted to address this heterogeneity by reporting LC rates for clinically relevant subgroups, including re-irradiated, ultracentral, and ≥ 4 cm tumors, although the small sample size limited the power of more extensive subgroup analyses. Our assessment of multiple variables in a relatively small cohort, with few events and strong competing risks evaluated over a long study period, increases the risk of overfitting despite our use of competing risk methodology. The multi-institutional nature of this study similarly introduces the possibility of other confounding variables affecting our results. Our data was derived from patients treated at 14 different proton therapy centers in the United States, which raises the possibility of unreported organization-specific practices influencing our findings. These factors limit the generalizability of our findings and underscore the need for larger studies with more homogenous patient populations, standardized treatment techniques, and prospective clinical trial design (ie. PBT versus photon SBRT/HFRT for de novo, early-stage central/ultracentral NSCLC).

To our knowledge, these data describe the largest prospectively enrolled sample of patients receiving IMPT delivered via SBRT or HFRT techniques for lung cancer. It is also the first prospectively enrolled, multi-center study evaluating PBT SBRT or HFRT for lung cancer that includes patients with SUNSET-defined ultracentrally located tumors or prior in-field radiation. At a minimum, we believe our series has demonstrated that PBT for SBRT/HFRT is a feasible treatment approach with a low acute toxicity rate for selected, high-risk lung cases. PBT should be considered as an alternative to photon-based techniques when OAR constraints limit the ability to achieve appropriate target coverage or when potential treatment-related morbidity can be reduced via dosimetric advantages. However, our cohort's limited follow-up duration restricts conclusions regarding the late safety outcomes of proton SBRT/HFRT. Longer follow-up is ultimately needed to validate the safety and durability of ablative PBT techniques for lung tumors. Despite the highly selected and heterogeneous nature of this cohort, along with limited survival and follow-up durations, these data provide a clinically relevant real-world benchmark for the use of PBT in challenging lung cancer scenarios.

## Other presentations

Portions of this manuscript were presented as an oral presentation at the 2025 Annual PTCOG Conference in Buenos Aires, Argentina, on 6/4/2025, and as an oral presentation at the 11th Annual PTCOG-NA Conference in Shreveport, Louisiana on 11/07/2025.

## Financial Support/Funding

None.

## CRediT authorship contribution statement

**Alexander J. Allen:** Writing – review & editing, Writing – original draft, Visualization, Validation, Software, Project administration, Methodology, Investigation, Formal analysis, Data curation, Conceptualization. **Charles B. Simone:** Writing – review & editing, Supervision, Resources, Methodology, Conceptualization. **Kai Sun:** Writing – review & editing, Validation, Software, Formal analysis. **David F. Alicia:** Writing – review & editing, Software, Data curation. **Ajay Banskota:** Writing – review & editing, Software, Data curation. **Gopal K. Bajaj:** Writing – review & editing, Resources. **John H. Chang:** Writing – review & editing, Resources. **James R. Gray:** Writing – review & editing, Resources. **Ryan S. Grover:** Writing – review & editing, Resources. **Christian C. Hyde:** Writing – review & editing, Resources. **Jenna B. Jatczak:** Writing – review & editing, Software, Data curation. **Kara E. Lehman:** Writing – review & editing, Software, Data curation. **Mark W. McDonald:** Writing – review & editing, Resources. **Stephen A. Mihalcik:** Writing – review & editing, Resources. **Sina Mossahebi:** Writing – review & editing, Software, Data curation. **Michelle R. Mundis Myers:** Writing – review & editing, Software, Data curation. **Yosan G. Okbamichael:** Writing – review & editing, Software, Data curation. **Stephanie M. Perkins:** Writing – review & editing, Resources. **Robert H. Press:** Writing – review & editing, Resources. **Zaker H. Rana:** Writing – review & editing, Resources. **Lane R. Rosen:** Writing – review & editing, Resources. **Ronny L. Rotondo:** Writing – review & editing, Resources. **Christopher C. Sinesi:** Writing – review & editing, Resources. **Henry K. Tsai:** Writing – review & editing, Resources. **James J. Urbanic:** Writing – review & editing, Resources. **Carlos E. Vargas:** Writing – review & editing, Resources. **Nathan Y. Yu:** Writing – review & editing, Resources. **Jing Zeng:** Writing – review & editing, Resources. **Matthew J. Ferris:** Writing – review & editing, Supervision, Resources, Project administration, Methodology, Conceptualization.

## Declaration of competing interest

The authors declare that they have no known competing financial interests or personal relationships that could have appeared to influence the work reported in this paper.

## Data Availability

Deidentified outcomes data will be shared upon reasonable request immediately following publication. Data can be obtained by contacting the corresponding author (Alexander Allen ajallen583@gmail.com).
