# Supplementary material for: Hypofractionated and stereotactic body proton radiotherapy for a high-risk lung cancer cohort: A multi-institutional experience from the proton collaborative group prospective registry
Source: Clin Transl Radiat Oncol. 2026 Sep 15;62:101291. doi: 10.1016/j.ctro.2026.101291 (PMC13602215; doi:10.1016/j.ctro.2026.101291)
Supplement: Supplementary file 1 [file mmc1.docx]

| **Supplemental Table 1. Toxicity Incidence by Proton Beam Therapy Modality** | | | |
| --- | --- | --- | --- |
| Toxicity | Intensity-Modulated Proton Therapy | Passive Scatter | p-value |
| Cough  G1  G2 | 33 (41.8%)  25 (31.7%)  6 (7.6%) | 11 (36.7%)  8 (26.7%)  2 (6.7%) | 0.628  0.964  0.868 |
| Dyspnea  G1  G2 | 26 (32.9%)  13 (16.5%)  9 (11.4%) | 12 (40%)  4 (13.3%)  5 (16.7%) | 0.488  0.688  0.462 |
| Radiation Dermatitis  G1  G2 | 9 (11.4%)  8 (10.1%)  1 (1.3%) | 10 (33.3%)  9 (30%)  0 (0%) | **0.007**  **0.011**  0.536 |
| Dysphagia  G1  G2 | 5 (6.3%)  5 (6.3%)  0 (0%) | 1 (3.3%)  0 (0%)  1 (3.3%) | 0.540  0.158  0.103 |
| Esophagitis  G1  G2 | 3 (3.8%)  2 (2.5%)  1 (1.3%) | 3 (10.0%)  2 (6.67%)  1 (3.3%) | 0.205  0.305  0.473 |
| Fatigue  G1  G2 | 24 (30.4%)  23 (29.1%)  1 (1.3%) | 11 (36.7%)  7 (23.3%)  3 (10%) | 0.530  0.546  **0.030** |
| Laryngeal Hemorrhage  G1  G2 | 0 (0%)  0 (0%)  0 (0%) | 1 (3.3%)  1 (3.3%)  0 (0%) | 0.103  0.103 |
| Hoarseness  G1  G2 | 1 (1.3%)  0 (0%)  1 (1.3%) | 4 (13.3%)  4 (13.3%)  0 (0%) | **0.007**  **0.001**  0.536 |
| Chest Wall Pain  G1  G2 | 2 (2.5%)  2 (2.5%)  0 (0%) | 1 (3.3%)  1 (3.3%)  0 (0%) | 0.8194  0.819 |
| Radiation Pneumonitis  G1  G2 | 2 (2.5%)  1 (1.3%)  2 (2.5%) | 0 (0%)  0 (0%)  0 (0%) | 0.379  0.536  0.379 |
